# Genetic mapping, marker development, and identification of candidate genes for powdery mildew resistance in *Malus baccata* ‘Jackii’

**DOI:** 10.3389/fpls.2025.1716290

**Published:** 2026-02-12

**Authors:** Matthias Pfeifer, Leonard Kurzweg, Buist Muçaj, Tom Burkhardt, Andreas Peil, Henryk Flachowsky, Ofere Francis Emeriewen, Thomas Wolfgang Wöhner

**Affiliations:** 1 Julius Kühn-Institut (JKI) - Federal Research Centre for Cultivated Plants, Institute for Breeding Research on Fruit Crops, Dresden-Pillnitz, Germany; 2 Institute of Plant Genetics, Department of Molecular Plant Breeding, Leibniz University Hannover, Hannover, Germany; 3 Faculty of Biology, Technical University of Dresden, Dresden, Germany; 4 Institute of Agricultural and Nutritional Sciences, Martin Luther University Halle-Wittenberg, Halle (Saale), Germany; 5 Faculty of Agriculture/Environment/Chemistry, University of Applied Sciences, Dresden, Germany

**Keywords:** apple, disease resistance, molecular markers, plant breeding, powdery mildew, QTL mapping, resistance gene

## Abstract

Powdery mildew, caused by *Podosphaera leucotricha*, is one of the most important fungal diseases in apple cultivation worldwide. *Malus baccata* ‘Jackii’, however, exhibits resistance to this pathogen, and a previous study demonstrated that this resistance, designated *Plbj*, co-segregates with an AFLP marker on linkage group 10. The objectives of this study were to construct genetic linkage maps, develop molecular markers, and identify candidate genes associated with this powdery mildew resistance, making use of the *M. baccata* ‘Jackii’ genome sequence. An F_1_ population derived from the cross ‘Idared’ × *M. baccata* ‘Jackii’ was phenotyped from 2009–11 and 2023–24 and genotyped with SNPs generated from tGBS and SSRs. Genetic linkage maps of *M. baccata* ‘Jackii’ were constructed, and QTL mapping confirmed the presence of the powdery mildew resistance locus *Plbj* on linkage group 10. In addition, a minor QTL was detected on linkage group five. Closely linked SSR and KASP markers were developed, and the *Plbj* locus was delimited to a 3,287,286-bp region on haplotype one of the *M. baccata* ‘Jackii’ genome, in which resistance gene candidates were identified. This study supports the direct application of molecular markers in breeding programmes and provides an essential background for future functional studies of *Plbj*.

## Introduction

The domesticated apple (*Malus domestica* Borkh.) is an important temperate fruit crop that faces multiple biotic stresses, and changes in climate and pathogen populations may further increase disease impact (Hanke et al., 2020; Strickland et al., 2021). Among the most significant pathogens is powdery mildew, caused by species of the genus *Podosphaera*. These fungi are highly adaptable and can infect over 10,000 plant species with frequent reports of host jumps and host range expansions (Kusch et al., 2024). Consequently, a perpetual race emerges between the fungus and plant breeders, who must persistently monitor and seek novel resistances. *Podosphaera leucotricha* (Ellis & Everh.), the primary powdery mildew pathogen of apple, has also been observed on other hosts such as *Photinia × fraserii*, *Prunus africana* and *Pyrus calleryana* (Mwanza et al., 2001; Garibaldi et al., 2005; Minnis et al., 2010). This obligate biotrophic, ascomycetous, heterothallic, ectoparasitic fungus causes considerable economic losses and increases production costs in apple cultivation each year (Coyier, 1974; Yoder, 2000; Takamatsu, 2013). The mycelium overwinters inside buds and, after bud burst, initiates primary infections, which cause delayed growth and deformations of shoots, leaves, flowers and fruits (Strickland et al., 2021). Sexual reproduction via ascospores is possible but appears to play a minor role in disease spread, whereas the formation of asexual conidia can drive multiple secondary infection cycles within a single season (Strickland et al., 2021). Management strategies to prevent yield losses include crop cultural practices, as well as chemical and biological measures (Strickland et al., 2021). The fungicides currently used in apple cultivation remain effective against the pathogen (Strickland et al., 2023). Their use in consistent rotation is essential to prevent resistance development (Strickland et al., 2023), especially since the emergence of fungicide resistance in powdery mildew has been frequently observed (Lesemann et al., 2006; Vielba-Fernández et al., 2020). The most desirable and environmentally sustainable strategy to control apple powdery mildew is the growing of resistant cultivars. This approach can also make apple production more economical by reducing both the risk of yield losses and the costs of control measures.

Various major resistances to powdery mildew, defined as single genes or clusters of genes with large effects providing qualitative resistance, are present in the genus *Malus*. *Pl-1* and *Pl-2* were identified in *Malus* × *robusta* and *Malus* × *zumi* (Knight and Alston, 1968), *Pl-w* in ‘White Angel’ (Gallott et al., 1985), *Pl-d* in accession D12 (Visser and Verhaegh, 1980), *Pl-m* in Mildew Immune Selection (Dayton, 1977; Bus et al., 2010) and *Plbj* in *Malus baccata* ‘Jackii’ (Dunemann and Schuster, 2009). Furthermore, powdery mildew resistance has also been reported in the apple clone U 211 (Stankiewicz-Kosyl et al., 2005) and in *Malus florentina* and *Malus sieboldii* (Schuster, 2000). Moreover, the knock-down of the expression of *Mildew Locus 0* (*MLO*) genes could have the potential to make apple trees more resistant to powdery mildew, though further research is required to fully understand the mechanisms involved (Pessina et al., 2016, 2017). As observed in apple, powdery mildew resistance genes can be overcome, as shown for *Pl-1* (Krieghoff, 1995; Kellerhals et al., 2013) and *Pl-2* (Caffier and Laurens, 2005; Caffier and Parisi, 2007). Therefore, the combination (pyramiding) of different powdery mildew resistance genes in a cultivar should be the objective, as this has the potential to increase the durability of the resistance (Mundt, 2018). Consequently, it remains of great importance not only to identify additional resistance genes, but also to facilitate the utilisation of existing ones. A previous study reported the presence of the *Plbj* resistance locus on linkage group (LG) 10 in *M. baccata* ‘Jackii’ (Dunemann and Schuster, 2009). The primary objectives of this study were to generate a higher-resolution map of LG 10, further narrow down the genetic region containing the *Plbj* resistance locus, and verify whether *Plbj* is still effective in our experimental field. Therefore, offspring from a cross between ‘Idared’ and *M. baccata* ‘Jackii’ (hereafter referred to as *Mb*j) were genotyped using various marker systems and phenotyped for susceptibility to powdery mildew over several years in the experimental field at the Julius Kühn-Institut (JKI) in Dresden-Pillnitz, Germany. An additional aim was to develop molecular markers tightly linked to *Plbj* for application in marker-assisted selection and to identify resistance gene candidates using the haplotype-resolved genome sequence of *Mb*j (Pfeifer et al., 2026).

## Materials and methods

### Plant material

**Figure 1:**
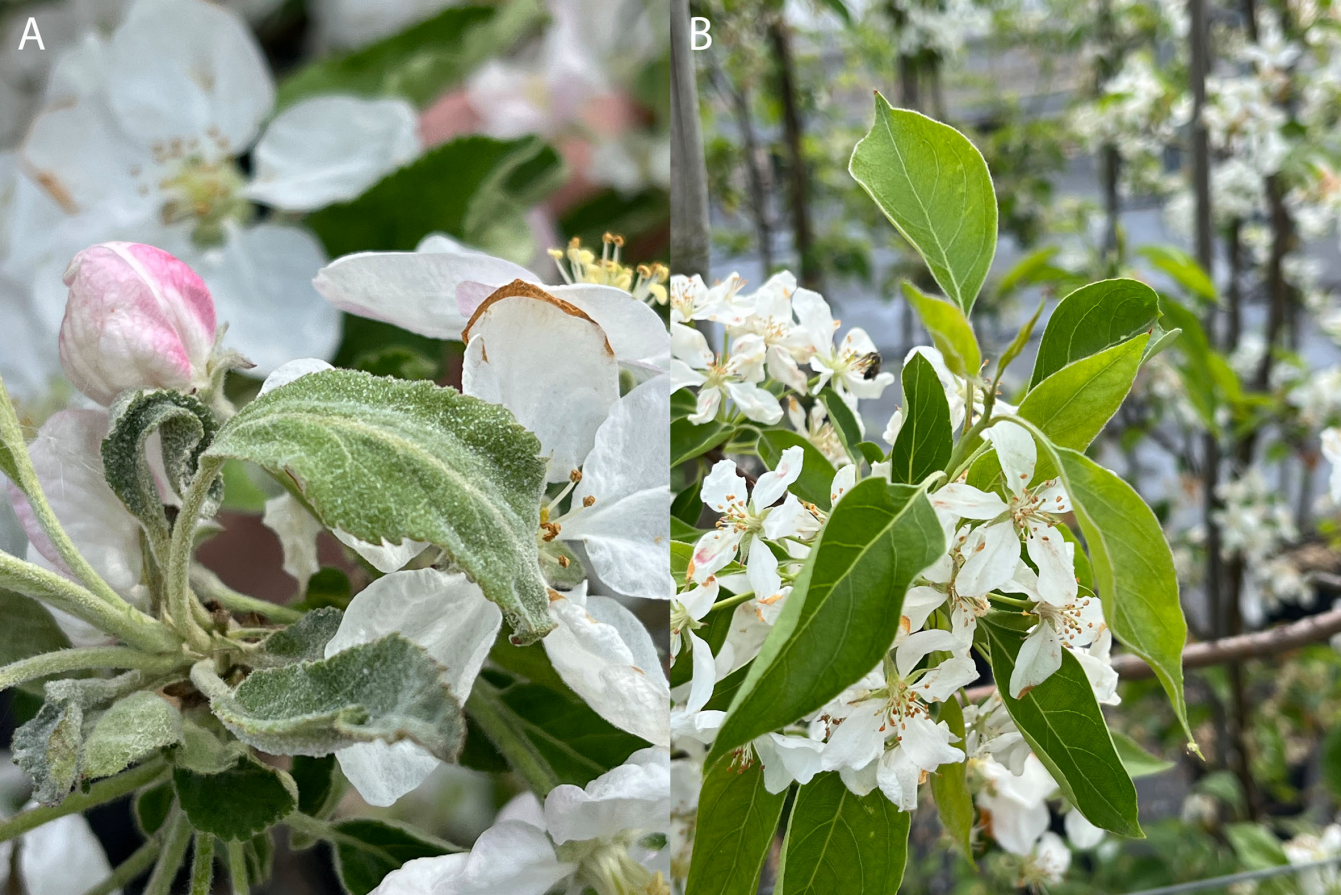
Symptoms of powdery mildew on ‘Idared’ **(A)**, and absence of symptoms on *M. baccata* ‘Jackii’ **(B)**.

The apple cultivar ‘Idared’ and the apple genotype *Mb*j are susceptible and resistant to powdery mildew, respectively (**Figure 1**). A cross between ‘Idared’ and *Mb*j resulted in 122 F_1_ individuals (designated 05225 and 06228 genotypes), which served as the primary mapping population for this study. These progenies are cultivated in the experimental field of the Julius Kühn-Institut (JKI) in Dresden-Pillnitz, Germany, without fungicide protection. Additionally, our study included a recently developed secondary population consisting of 127 individuals from the same cross combination (designated 24230 genotypes), as well as 45 seedlings derived from *Mb*j pollinated by F_1_ individuals of ‘Idared’ × *Mb*j (designated Jackii-OA), all of which are maintained under greenhouse conditions without fungicide protection.

### Powdery mildew phenotyping

Phenotyping of natural powdery mildew infestation was conducted for the primary mapping population (05225 and 06228 genotypes) in the field during the spring and summer of 2009, 2010, 2011 and 2023. In 2024, assessments were performed only in spring, as the trees were pruned afterwards and the prevalence of powdery mildew post-pruning was too low for a reliable evaluation. **Table 1** shows the phenotyping scales for the assessment of powdery mildew infestation. The scale applied in 2023 and 2024 was published by Lateur et al. (2022). For 2009-11, plants with powdery mildew infestation scores of zero to three were classified as resistant, whereas those with scores of four to nine were classified as susceptible. For 2023-24, plants with a score of one were considered resistant, while those with scores from two to nine were considered susceptible. Plants grown in the greenhouse (24230 and Jackii-OA genotypes) were classified only as either resistant or susceptible, without any intermediate categories, based on the presence or absence of disease symptoms resulting from natural infection.

**Table 1:** Used phenotyping scales for powdery mildew infection.

Score	Phenotyping scale 2009-11	Phenotyping scale 2023-24 (Lateur et al., 2022)
0	No visible symptom	not applicable
1	Very few sporulating dots	No visible symptom (0%)
2	Very few to few sporulating dots	One or very few organs affected, detectable on close scrutiny of the tree (0-1%)
3	Up to 25% of the tree affected by infected leaves/shoots	Infected organs readily apparent but without important consequences for the tree (1-5%)
4	Intermediate rating	Intermediate rating
5	Up to 50% of the tree affected by infected leaves/shoots	Primary mildew widespread over the branches, inducing the infection of a substantial part of the crown (± 25%)
6	Intermediate rating	Intermediate rating
7	Up to 75% of the tree affected by infected leaves/shoots	Heavy infection; half of the organs are badly affected (± 50%)
8	Intermediate rating	Intermediate rating
9	Up to 100% of the tree affected by infected leaves/shoots	Crown completely affected, nearly all top of the organs are infected (> 90%)

### tGBS genotyping and SNP identification

Young leaves of the 122 F_1_ individuals of the primary mapping population along with four replicates of both parents were harvested, lyophilised, and sent to Data2Bio (Ames, IA, USA) for DNA extraction and tunable genotyping-by-sequencing (tGBS) analysis (Ott et al., 2017) using the restriction enzyme *Bsp*1286I and an Illumina HiSeq X instrument (Illumina, Inc., San Diego, CA, USA), according to the company’s specifications. Single-nucleotide polymorphism (SNP) identification and tGBS genotyping were performed as described by Pfeifer et al. (2026). Briefly, quality-trimmed sequence reads, excluding regions with a PHRED score ≤ 15, were aligned to haplotype 1 (HT1) of the *Mb*j genome using GSNAP (Wu and Nacu, 2010). Only confidently mapped reads that aligned to a unique location in HT1 were used for SNP identification. For homozygous SNPs, the most common allele had to be supported by at least 80% of all aligned reads at a given position and confirmed by a minimum of five unique reads. For heterozygous SNPs, the two most common alleles each had to be supported by at least 30% of the aligned reads at that position and confirmed by at least five unique reads. For tGBS genotyping, additional criteria were applied: homozygous calls required at least five supporting reads, with ≥ 90% of aligned reads carrying the same nucleotide at that site; heterozygous calls required each allele to be supported by at least two reads, with a combined minimum of five reads, each allele representing > 20% and both alleles together ≥ 90% of all reads at that site. The minimum calling rate for SNPs across the population was set to ≥ 50%, and the minor allele frequency had to be ≥ 10%. Finally, SNPs lacking a sufficient number of reads to make genotype calls were imputed using Beagle v5.4 (Browning et al., 2018). Similar empirical parameters, which aim to minimise false positive and false negative SNP calls, have already been applied in wild *Malus* species (Emeriewen et al., 2020) and other plants (Li et al., 2018; Zheng et al., 2018).

### SSR marker sourcing, development and genotyping

Since tGBS-derived SNPs are not easily transferable to other sample sets, we sourced SSR markers from the literature to serve as anchor markers for the construction of genetic linkage maps (Hokanson et al., 1998; Liebhard et al., 2002; Yamamoto et al., 2002a, 2002; Hemmat et al., 2003; Vinatzer et al., 2004; Silfverberg-Dilworth et al., 2006; Celton et al., 2009; Emeriewen et al., 2014, 2017). Initially, 115 SSRs were selected to be distributed across all 17 chromosomes of *Malus*, from the HiDRAS website (2025) and tested for polymorphism in the parents ‘Idared’ and *Mb*j and a subset of six offspring. Of the 71 SSRs showing polymorphisms in *Mb*j, a total of 63 were used in this study (**Supplementary Table S1**). In addition, seven new SSRs were developed to flank the resistance loci. Therefore, SSR motifs were searched for in the genome sequences of GDDH13 v1.1 (Daccord et al., 2017) and *Mb*j (Pfeifer et al., 2026), in the regions identified by preliminary QTL mapping, and primer pairs (**Supplementary Table S2**) were designed using Primer3web v4.1.0 (Koressaar and Remm, 2007; Untergasser et al., 2012). The 122 F_1_ individuals (05225 and 06228 genotypes) grown in the field and both parents were genotyped with the 63 SSRs selected from the HiDRAS website (**Supplementary Table S1**) and the seven newly developed SSR markers (**Supplementary Table S2**). The 127 F_1_ individuals (24230 genotypes) and the 45 *Mb*j seedlings (Jackii-OA genotypes), both cultivated in the greenhouse, were genotyped with five of the seven newly developed SSR markers. These five markers, which were linked to *Plbj*, were used to validate their association with resistance and to delimit the *Plbj* locus.

### DNA isolation, PCR and fragment analysis

DNA was extracted from leaves using the DNeasy Plant Mini Kit (Qiagen, Hilden, Germany) according to the manufacturer’s protocol. DNA quantification was performed with the NanoDrop One^C^ spectrophotometer (Thermo Fisher Scientific Inc., Waltham, MA, USA). Multiplex-PCR was conducted using the Type-it Microsatellite PCR Kit (Qiagen, Hilden, Germany). The PCR reaction mix consisted of 1 μl primer or primer mix (each primer at a concentration of 1 pmol/µl), 1 μl ddH_2_O, 5 μl Type-it Multiplex PCR Master Mix, 1 μl Q-solution, and 2 μl DNA (10 ng/μl). The PCR conditions were as follows: initial denaturation at 95 °C for 5 minutes, followed by 32 cycles of 95 °C for 1 minute, 60 °C for 1 minute and 30 seconds, and 72 °C for 1 minute, with a final elongation step at 60 °C for 30 minutes. The PCR products were then diluted 1:100 with ddH_2_O, and 1μl of this dilution was mixed with 9 μl of ABI-solution (a mixture of 1 ml Hi-Di formamide and 6 μl GeneScan-600 LIZ size standard, both from Applied Biosystems, Waltham, MA, USA). The samples were then denatured at 95 °C for 5 minutes prior to analysis using the Applied Biosystems 3500xL Genetic Analyzer (Applied Biosystems, Waltham, MA, USA). GeneMapper Software v6 (Applied Biosystems, Waltham, MA, USA) was used to visualise and analyse the SSR alleles.

### Construction of genetic linkage maps

Prior to genetic linkage map construction, the 324,420 non-imputed SNP markers for the *Mb*j HT1 were filtered. In the first step, all SNP markers that did not yield identical results across all four replicates of ‘Idared’ and *Mb*j, were excluded. SNP markers with more than 10% missing values in the progeny were also excluded. For hk×hk and nn×np markers, chi-square values were calculated, and only markers with values below 10 were retained. For each linkage group, SNPs were ranked by physical inter-marker distance and all markers with inter-marker distances of 100 kb or greater were included and at least 120 markers per group were selected. From the selected SNPs, the imputed genotypic data along with the 70 SSRs were used for the construction of the genetic map of *Mb*j using JoinMap 5 (Van Ooijen, 2018). The final map of *Mb*j was calculated after excluding identical SNP markers, followed by manually correcting implausible double-recombinations, using the regression mapping algorithm and Kosambi’s mapping function. Linkage maps were visualised using MapChart (Voorrips, 2002).

### QTL analysis

The SNP and SSR genotypic data of the primary F_1_ mapping population (05225 and 06228 genotypes), the final genetic map of *Mb*j together with multi-year phenotypic datasets, were used to determine genotype-phenotype associations and conduct QTL analysis using MapQTL 5 (Van Ooijen, 2004). Kruskal-Wallis analysis was applied to identify markers significantly associated with the phenotypic data, whereas interval mapping was used to localise the corresponding QTL intervals. A permutation test at a 95% confidence level was conducted to assess the significance of identified QTLs by determining the LOD threshold at both the genome-wide and chromosome-wide levels (Van Ooijen, 2004).

### Mapping resistance as a single qualitative trait

To map resistance as a single qualitative trait, named *Plbj*, phenotypic data from 2023–24 for the 122 F_1_ individuals in the field (05225 and 06228 genotypes) were transformed for each individual into resistant (score one) or susceptible (scores two to nine). These data were then added to the other molecular markers on LG 10, and the genetic map was calculated using JoinMap 5 (Van Ooijen, 2018).

### Assigning resistance-linked markers to haplotypes of *Mb*j

To identify the resistance-associated haplotypes of *Plbj* and the minor QTL on LG 5, two independent approaches were applied. In the first approach, the primer sequences of the newly developed SSR markers were aligned to the genome sequences of both haplotypes of *Mb*j (Pfeifer et al., 2026) using the Basic Local Alignment Search Tool (BLAST; Altschul et al., 1990) and CLC Main Workbench 25.0 (Qiagen, Venlo, Netherlands). Expected PCR product sizes in base pairs were calculated by considering the distance between the outermost primer positions in the haplotypes and accounting for any additional bases present in the primers but absent from the assembled genome. The expected PCR product sizes were then compared with the fragment sizes observed in the fragment length analysis. In the second approach, the alleles of nn×np SNP markers from both parents and the primary mapping population (122 F_1_ individuals) were examined. Based on the inheritance patterns and the assumption that *Mb*j is the resistance donor, the SNP alleles associated with resistance were identified. In both approaches, resistance-associated markers were determined by comparing the mean disease scores of the 122 F_1_ individuals for the respective allele combinations and assigning resistance to the markers associated with lower average disease severity. For the assignment of the minor QTL on LG 5, only individuals lacking *Plbj* were taken into account to avoid distortion of the disease score average caused by *Plbj* when identifying the resistance-associated markers.

### KASP marker development and genotyping

Based on the genetic linkage map, two Kompetitive Allele Specific PCR (KASP) markers were developed in proximity to *Plbj*. The SNP positions of these KASP markers are reflected in their names: KASP_HT1_LG10_20042456 and KASP_HT1_LG10_23962775. Primer sequences for these KASP markers are listed in **Supplementary Table S3**. KASP genotyping was performed using a reaction mix consisting of the KASP assay mix (a mixture of two allele-specific forward primers and one common reverse primer), KASP-TF V4.0 2× Master Mix (LGC Group, Teddington, England) and DNA. Reactions were run on a CFX96 Touch Real-Time PCR Detection System (Bio-Rad Laboratories, Inc., Hercules, CA, USA). The 10 µl KASP reaction contained 5 μl 2× KASP master mix, 0.14 μl KASP assay mix, 1 μl DNA (10 ng/μl) and 3.86 μl ddH_2_O. PCR was performed under the following conditions: an initial activation at 94 °C for 15 minutes, followed by 10 cycles of denaturation at 94 °C for 20 seconds and annealing/elongation for 1 minute with a temperature gradient from 61 to 55 °C (decreasing 0.6 °C per cycle), and then 26 cycles of 94 °C for 20 seconds and 55 °C for 1 minute and finally, a 1-minute step at 37 °C for the read stage. Data analysis was performed with CFX Manager v3.1 (Bio-Rad Laboratories, Inc., Hercules, CA, USA). DNA from the cultivars ‘Idared’, ‘Golden Delicious’, ‘Granny Smith’, ‘Delicious’, ‘Cox Orange’, ‘Jonathan’, ‘Mcintosh’, ‘Braeburn’, ‘Gala’ and *Mb*j were used as control for the validation of the KASP markers.

### Identification of resistance gene candidates in the genome of *Mb*j

Resistance gene candidates were identified based on annotation data published by Pfeifer et al. (2026). Genes that were annotated with an NB-ARC domain (PF00931), leucine-rich repeat N-terminal domain (PF08263), TIR domain (PF01582), Rx N-terminal domain (PF18052) or associated with the Gene Ontology terms defence response (GO:0006952), response to other organism (GO:0051707) or protein kinase activity (GO:0004672), as well as proteins whose names contained the term disease resistance, were considered the most likely resistance gene candidates.

## Results

### Phenotyping results of the ‘Idared’ × *Mb*j F_1_ populations

An overview of the phenotyping results is presented in **Table 2**. In the primary mapping population in the field (122 F_1_ individuals of ‘Idared’ × *Mb*j, designated 05225 and 06228) the highest mean powdery mildew score was observed in summer 2023 (2.76), whereas the lowest mean score occurred in spring 2010 (1.34). Maximum disease severity among the offspring ranged from seven to nine, indicating consistently high infection pressure in the field. Across all years, 71 individuals were classified as resistant, while 51 were scored as susceptible at least once. In 2023 and 2024, only 120 individuals could be evaluated due to the loss of two genotypes. Spearman correlation coefficients between field assessments ranged from 0.61 (summer 2010 vs. spring 2011) to 0.97 (spring 2023 vs. spring 2024). Phenotypic distributions consistently showed a right-skewed pattern. In the secondary F_1_ population (127 individuals, designated 24230) from the same cross combination, phenotyped in the greenhouse in 2025, 46 individuals were resistant and 81 were susceptible. Considering both populations, a total of 117 were classified as resistant and 132 as susceptible.

**Table 2:** Overview of phenotyping results from the ‘Idared’ × *M. baccata* ‘Jackii’ F_1_ populations.

Genotypes	Time point of powdery mildew assessment	Location	Mean score in offspring	Max. value in offspring	Resistant genotypes^a^	Susceptible genotypes^b^
05225 and 06228	Spring 2009	Field	1.89	9	94	28
	Summer 2009		2.32	9	81	41
	Spring 2010		1.34	7	109	13
	Summer 2010		1.51	7	109	13
	Spring 2011		1.82	7	103	19
	Summer 2011		2.40	7	92	30
	Spring 2023		2.08	7	75	45
	Summer 2023		2.76	9	71	49
	Spring 2024		2.20	8	74	46
24230	Summer 2025	Greenhouse	/	/	46	81

a Resistant genotypes: scores 0–3 in 2009-11, score 1 in 2023-24.

b Susceptible genotypes: scores 4–9 in 2009-11, scores 2–9 in 2023-24. Different phenotyping scales were applied in 2009–11 and 2023-24; in 2025 plants were only classified as either resistant or susceptible.

### Genetic linkage map of *Mb*j haplotype 1

A genetic linkage map was constructed for *Mb*j HT1, comprising 17 linkage groups with a total length of 1,071 centimorgans (cM). After quality trimming, 99.2% of the raw sequencing data (in bp) from the tGBS analysis were retained, of which 56.0% of the reads aligned uniquely to HT1. Application of all quality filters resulted in a set of 324,420 SNP markers. These SNPs were well distributed across all 17 chromosomes, with the lowest marker density observed on LG 14, which still contained 15,305 SNPs. During the construction of genetic linkage maps from the 324,420 non-imputed SNP markers, 261,000 were excluded because they were either homozygous in *Mbj* or inconsistent across the four ‘Idared’ or *Mbj* replicates. From the remaining 63,420 SNPs (19.6%), those with > 10% missing data in the progeny and chi-square values ≥ 10 were discarded, resulting in 29,245 SNP markers. From the imputed data of these markers, a total of 2,043 with the largest inter-marker distances were loaded into JoinMap 5, and after excluding identical SNP markers, this resulted in 948 SNP markers being distributed across the 17 linkage groups. Of these 948 SNP markers, 48 segregated in the hk×hk configuration and 900 in the nn×np configuration. Of the 63 simple sequence repeat (SSR) markers selected from the HiDRAS website (2025), 58 could be assigned to the 17 linkage groups, with three of them displaying multilocus alleles. Of the seven newly developed SSRs, five were mapped to LG 10, and one each to LG 5 and LG 7. **Supplementary Table S4** provides an overview of the number of markers per LG and their genetic lengths. **Supplementary Figure S1** shows the 17 linkage groups, including marker names and their respective positions in cM.

### QTL analysis reveals the major *Plbj* resistance locus and a novel minor locus

**Figure 2:**
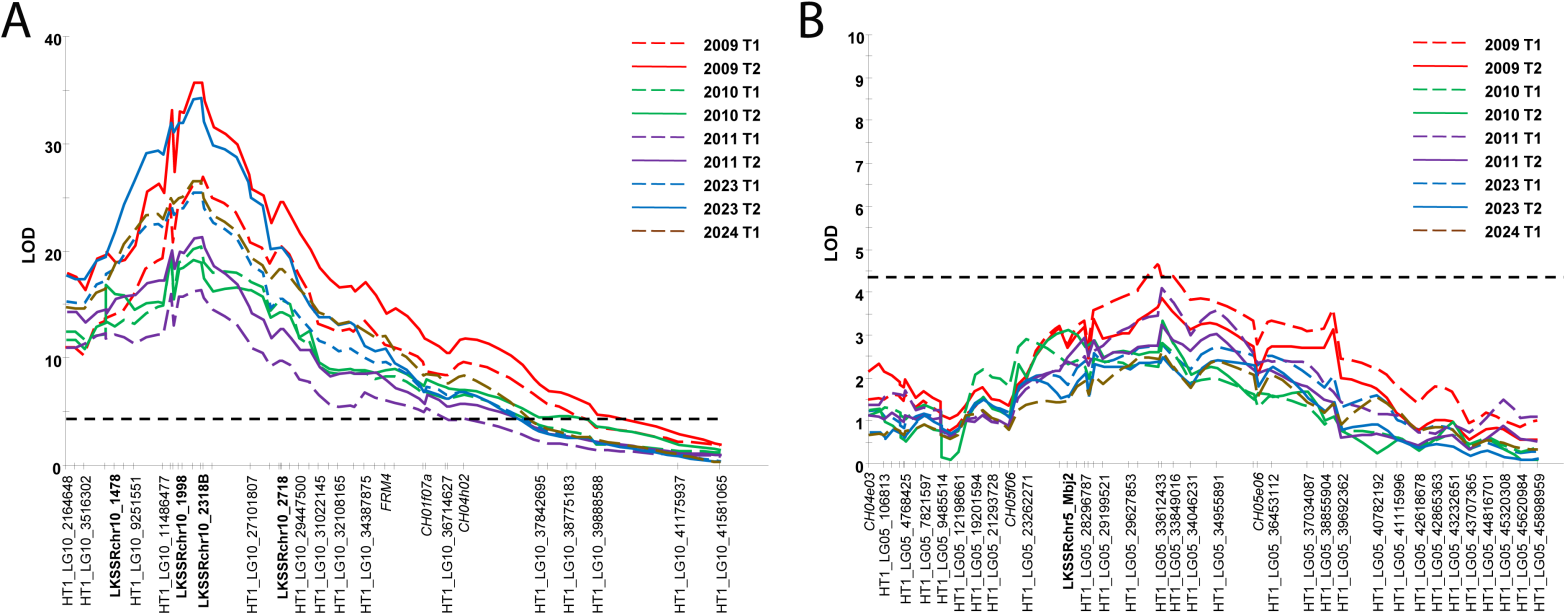
LOD score profiles of the identified QTLs on LG 10 **(A)** and 5 **(B)** of *M. baccata* ‘Jackii’, derived from powdery mildew infection data at various time points using interval mapping, indicating the approximate position of *Plbj.* T1: time point 1 (spring), T2: time point 2 (summer). The dotted line represents the genome-wide significance threshold. SSRs developed in this project are shown in bold and SSRs selected from the HiDRAS website (2025) in italics. For a better readability, only a subset of markers is presented.

Kruskal-Wallis (KW) analysis using the phenotypic data of the primary F_1_ mapping population, together with the constructed genetic linkage map, revealed a consistent and significant association of markers on LG 10 and LG 5 with resistance to powdery mildew across all the phenotyped time points in 2009, 2010, 2011, 2023, and 2024. The highest KW values for markers on LG 10 ranged from 51.15 to 109.04 (df = 1 or 3; significance level: *p* < 0.0001), whereas those for LG 5 ranged from 9.67 to 17.96 (df = 1; significance level: *p* < 0.005). Interval mapping confirmed significant associations on both LG 10 and 5, and **Table 3** presents the markers with the highest LOD scores on these linkage groups from this analysis. The major QTL on LG 10 showed LOD scores ranging from 16.34 to 35.73, while the minor QTL on LG 5 had scores between 2.49 and 4.63. A permutation test showed that the QTL on LG 10 consistently exceeded the genome-wide threshold, whereas the QTL on LG 5 surpassed the genome-wide significance threshold only with data from spring 2009. In spring 2011 and 2023, as well as in the summers of 2009-11, LOD scores on LG 5 only exceeded the chromosome-wide significance threshold. **Figure 2** shows the LOD score profiles for the identified QTLs on LG 10 and 5. Whereas the QTL on LG 10 explained up to 74% of the phenotypic variance, the QTL on LG 5 explained between 9.1% and 16.0% of the phenotypic variance. Newly developed SSR markers LKSSRchr10_1478 and LKSSRchr10_2718 were found to flank the QTL region on LG 10, with LKSSRchr10_1998 and LKSSRchr10_2318B being highly linked to the QTL peak. LKSSRchr5_Mbj2 was associated with resistance conferred by the minor QTL on LG 5.

**Table 3:** Markers with the highest LOD scores on LG 10 and 5 identified by interval mapping.

Time point of powdery mildew assessment	Linkage group	Marker with highest LOD score	LOD score	Explained variance (%)	Average phenotypic score with/without resistance allele	Significance with permutation test at a 95% confidence level
Spring 2009	10	HT1_LG10_25523367	26.92	63.8	0.39/3.92	Genome-wide
Summer 2009		LKSSRchr10_2318B	35.73	74.0	0.43/4.96	Genome-wide
Spring 2010		LKSSRchr10_2318B	20.39	53.7	0.19/2.88	Genome-wide
Summer 2010		LKSSRchr10_1978	19.87	52.8	0.43/2.84	Genome-wide
Spring 2011		LKSSRchr10_2318B	16.34	46.0	0.78/3.37	Genome-wide
Summer 2011		LKSSRchr10_2318B	21.24	55.1	1.22/4.22	Genome-wide
Spring 2023		LKSSRchr10_2318B	25.50	62.4	1.03/3.56	Genome-wide
Summer 2023		LKSSRchr10_2318B	34.27	73.2	1.00/5.28	Genome-wide
Spring 2024		LKSSRchr10_2318B	26.56	63.9	1.05/3.90	Genome-wide
Spring 2009	5	HT1_LG05_33612433	4.63	16.0	2.50/4.48^a^	Genome-wide
Summer 2009		HT1_LG05_31890599	3.85	13.5	3.93/5.42^a^	Chromosome-wide only
Spring 2010		HT1_LG05_23262271	2.92	10.5	2.13/3.27^a^	Not significant
Summer 2010		HT1_LG05_31890599	4.00	14.0	2.18/3.34^a^	Chromosome-wide only
Spring 2011		HT1_LG05_31890599	4.09	14.3	2.43/3.65^a^	Chromosome-wide only
Summer 2011		HT1_LG05_31890599	3.24	11.5	3.62/4.25^a^	Chromosome-wide only
Spring 2023		HT1_LG05_31890599	2.78	10.1	2.87/3.93^a^	Chromosome-wide only
Summer 2023		HT1_LG05_31890599	2.49	9.1	4.56/5.66^a^	Not significant
Spring 2024		HT1_LG05_31890599	2.62	9.6	3.12/4.24^a^	Not significant

a For LG 5 only offspring without *Plbj* were considered to avoid distortion in the average phenotypic score.

### *Plbj* is located between markers LKSSRchr10_1998 and LKSSRchr10_2318B

**Figure 3:**
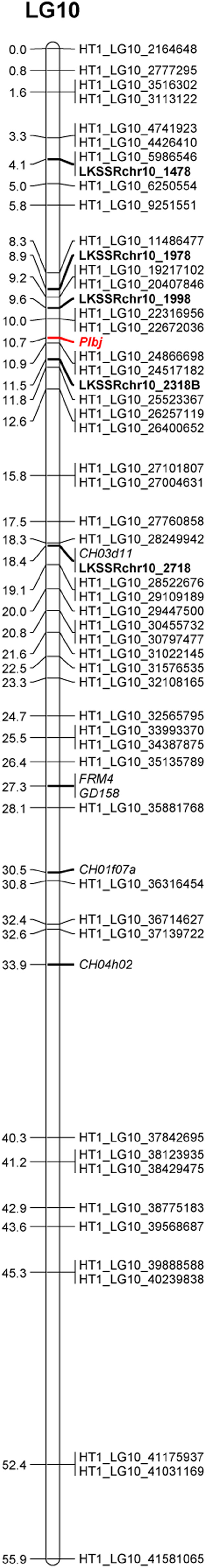
Genetic linkage map of LG 10 of haplotype 1 of *M. baccata* ‘Jackii’, showing the approximate position of *Plbj*. SSRs developed in this project are shown in bold, SSRs selected from the HiDRAS website (2025) in italics, and *Plbj* is indicated with a bold red italic label.

**Figure 4:**
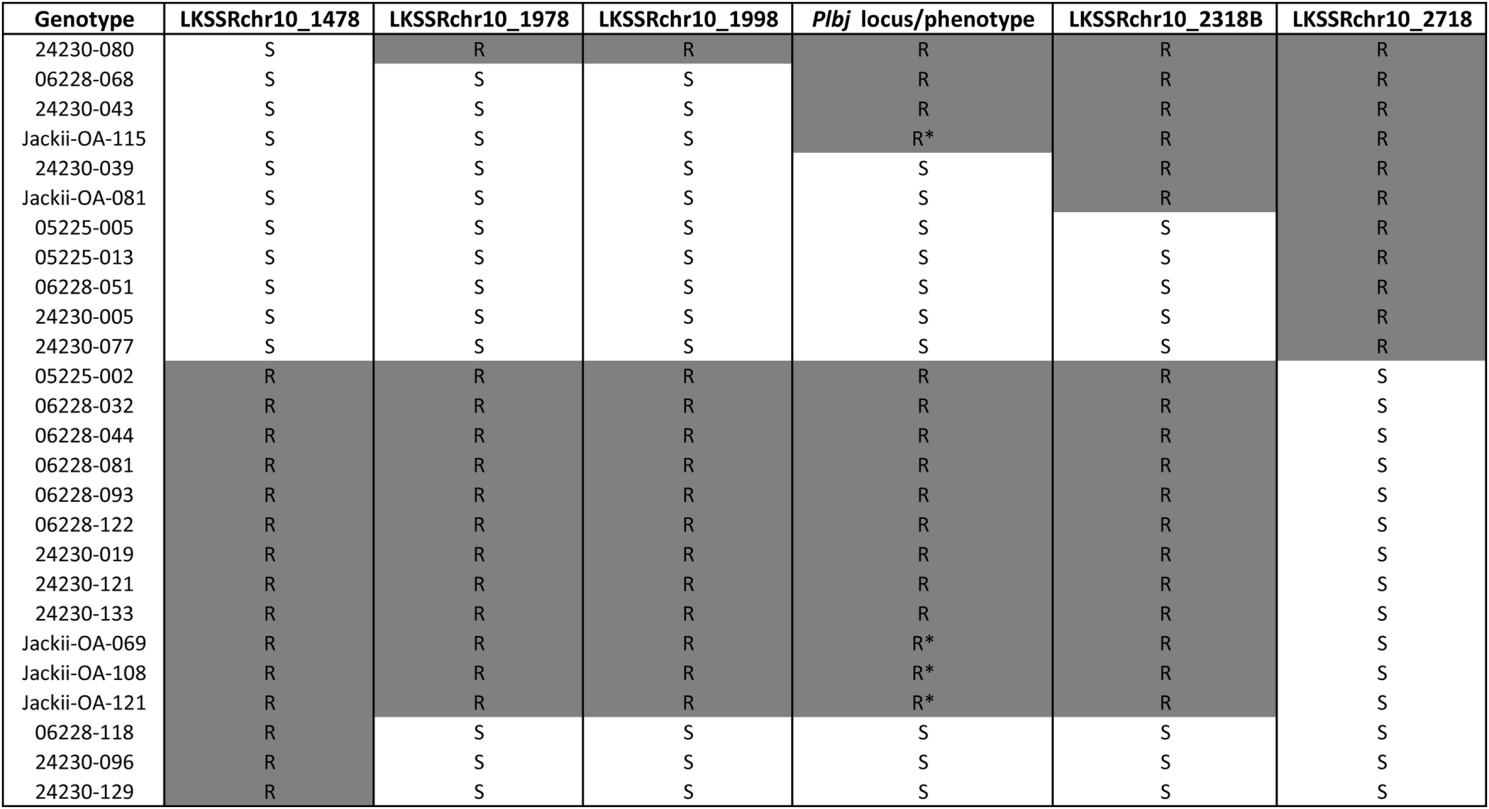
Graphical representation of the *Plbj* region based on SSR markers. Twenty-six individuals show recombination events between SSR markers LKSSRchr10_1478 and LKSSRchr10_2718, suggesting that *Plbj* is located between the SSR markers LKSSRchr10_1998 and LKSSRchr10_2318B. Genotypes 05225 and 06228 are recombinant individuals from the primary F_1_ mapping population maintained in the field, whereas genotypes 24230 (secondary F_1_ population) and Jackii-OA (*M. baccata* ‘Jackii’ seedlings pollinated by F_1_ individuals derived from ‘Idared’ × *M. baccata* ‘Jackii’) are maintained in the greenhouse. R: resistance allele, S: susceptibility allele. For the *Plbj* locus/phenotype R denotes a resistant and S denotes a susceptible phenotype; an asterisk indicates resistance presumed due to low powdery mildew pressure.

In the primary mapping population in the field (122 F_1_ individuals), all plants lacking the resistance-associated SSR allele of LKSSRchr10_2318B were phenotyped as susceptible to powdery mildew in the 2023–24 dataset, whereas only one individual carrying the resistance alleles of all five newly developed SSRs on LG 10 was also phenotyped as susceptible. Phenotyping in 2009–11 identified seven individuals carrying susceptibility alleles of these five SSRs that were nonetheless phenotyped as resistant. In the secondary population (127 greenhouse-grown F_1_ individuals), three individuals also showed genotype-phenotype incongruence as they carried only resistance-associated SSR alleles but were phenotyped as susceptible, whereas all individuals lacking resistance-associated marker alleles were also phenotyped as susceptible. Among the 45 *Mb*j seedlings (pollinated by F_1_ individuals derived from ‘Idared’ × *Mb*j), five individuals with recombinations between LKSSRchr10_1478 and LKSSRchr10_2718 were identified. One showed visible powdery mildew symptoms, while the remaining four were presumed resistant; however, confirmation under higher powdery mildew pressure is required. Mapping of *Plbj* as a single qualitative trait using the 2023–24 phenotypic dataset of the primary mapping population positioned the locus between SSR markers LKSSRchr10_1998 and LKSSRchr10_2318B (**Figure 3**). To validate this, 26 individuals with recombinations between LKSSRchr10_1478 and LKSSRchr10_2718 from different populations were examined. A comparison of these recombinants (**Figure 4**) revealed that genotypes possessing the resistance alleles of LKSSRchr10_1998 and LKSSRchr10_2318B exhibited a resistant phenotype, whereas genotypes lacking the resistance alleles at both markers exhibited a susceptible phenotype. In contrast, the presence of only one resistance allele at either of the two markers could result in either a resistant or a susceptible phenotype due to recombination events. Individuals 06228-068, 24230–043 and Jackii-OA-115 indicate that *Plbj* lies downstream of LKSSRchr10_1998, whereas 24230–039 and Jackii-OA-81 suggest it is upstream of LKSSRchr10_2318B. Taken together, these findings delimit *Plbj* to the interval between LKSSRchr10_1998 and LKSSRchr10_2318B.

### SNP and BLAST-supported SSR marker analysis identifies resistance-associated haplotypes

A comparison between the expected PCR product sizes of the newly developed SSRs and those observed in the fragment length analysis revealed differences of up to five bp. Nevertheless, the size differences between resistance- and susceptibility-associated alleles within each marker were consistent with the expected values. When phenotypic data were included, it became evident that *Plbj* is linked to the marker alleles assigned to HT1. In contrast, lower average disease scores in individuals lacking *Plbj* were associated with allele 111 from LKSSRchr5_Mbj2, indicating that the minor QTL on LG 5 is located on HT2. SSR LKSSRchr10_1438, designed for LG 10 based on the GDDH13 genome (Daccord et al., 2017), amplified fragments that were not linked to powdery mildew resistance and instead mapped to LG 7 in *Mb*j. An overview of the results for the six newly developed SSRs linked to powdery mildew resistance is provided in **Supplementary Table S5**. The assignment of *Plbj* and the minor QTL on LG 5 to their respective haplotypes was further confirmed by examining selected SNP markers linked to the two resistance loci. Based on the known parental alleles and the segregation pattern in the F_1_ progeny, the resistance-associated SNPs contributed by *Mb*j could be assigned to the corresponding haplotypes. As shown in **Table 4**, *Plbj* is located on HT1, while the minor QTL on LG 5 is located on HT2.

**Table 4:** SNP marker-based identification of resistance-associated haplotypes in *M. baccata* ‘Jackii’.

Marker	SNP-alleles in ‘Idared’	SNP-allele in *M. baccata* ‘Jackii’ haplotype 1	SNP-allele in *M. baccata* ‘Jackii’ haplotype 2	Resistance-associated SNP-allele combination in offspring	Resistance-associated SNP-allele in *M. baccata* ‘Jackii’
HT1_LG10_22316956	GG	C	G	CG	C
HT1_LG10_24866698	CC	T	C	TC	T
HT1_LG10_25523367	TT	T	C	TT	T
HT1_LG05_29627853	GG	A	G	GG^a^	G
HT1_LG05_33612433	AA	A	T	AT^a^	T
HT1_LG05_33849016	AA	G	A	AA^a^	A

a For LG 5 only offspring without *Plbj* were considered to avoid distortion in identifying the resistance-associated SNP-allele combination.

### Validation of KASP markers through comparison to SSR markers

The newly developed KASP markers KASP_HT1_LG10_20042456 and KASP_HT1_LG10_23962775, located between LKSSRchr10_1478 and LKSSRchr10_1978, and between LKSSRchr10_1998 and LKSSRchr10_2318B, respectively, showed complete concordance with the SSR marker data. No apparent double recombination events were observed between the flanking SSR markers in any individual of the primary F_1_ mapping population. Specifically, for KASP_HT1_LG10_20042456, the resistance-associated allele was consistently linked to the T_Hex_ allele, and for KASP_HT1_LG10_23962775, resistance was associated with the G_Hex_ allele. Moreover, the KASP-assays were tested on nine founders of apple ‘Idared’, ‘Golden Delicious’, ‘Granny Smith’, ‘Delicious’, ‘Cox Orange’, ‘Jonathan’, ‘Mcintosh’, ‘Braeburn’ and ‘Gala’ and all of them consistently exhibited the FAM-labeled C-allele linked to susceptibility to powdery mildew in *Mb*j.

### Resistance gene candidates within the *Plbj* locus on HT1

The region of interest, defined as the interval between markers LKSSRchr10_1998 and LKSSRchr10_2318B on HT1 (specifically from position 21,391,236 to 24,678,521 bp, spanning 3,287,286 bp), contains a total of 209 annotated genes (**Supplementary Table S6**). Among these, 29 genes were identified as the most likely resistance gene candidates (**Supplementary Table S7**).

## Discussion

Powdery mildew is one of the most important fungal diseases in apple, and its impact may further increase in the future (Strickland et al., 2021; García-Gómez et al., 2024). At the same time, demand for reduced pesticide use is steadily growing, making genetic host resistance increasingly important in sustainable apple production. Pyramiding different resistance genes not only has the potential to increase durability but can even reduce disease severity beyond that of the single most effective resistance gene (Mundt, 2018). Consequently, substantial efforts in apple breeding aim to combine high fruit quality with multiple resistance genes targeting either the same pathogen or different diseases simultaneously (Kellerhals et al., 2013; Baumgartner et al., 2015). For powdery mildew, genotypes carrying *Pl-1* and *Pl-2* have been bred (Kellerhals et al., 2013). However, both of these resistance genes have already been overcome when deployed individually (Krieghoff, 1995; Caffier and Laurens, 2005; Caffier and Parisi, 2007; Kellerhals et al., 2013). This highlights the urgent need to incorporate additional, more robust sources of powdery mildew resistance into apple cultivars.

In the present study, a genetic linkage map of *Mb*j HT1 was generated, spanning a total length of 1,071 cM, which is comparable to previously published genetic linkage maps in apple (Celton et al., 2009; Norelli et al., 2017; Emeriewen et al., 2020). Only the genetic linkage map of *Mb*j HT1 was constructed, because QTL mapping performed using a preliminary map of HT2 yielded similar results (not shown). This outcome was expected given the stringent SNP filtering criteria: for heterozygous SNPs, which serve as the informative markers for genetic mapping, high read support (≥ 90% of all reads at a given site supporting the two alleles) and unique alignment to HT1 indicated that the alternative allele resides on the complementary haplotype (HT2). Most of the SNP markers on the genetic linkage map of *Mb*j HT1 were ordered consistently with their physical positions, supporting the overall accuracy of the map. To complement the SNP-based map, SSR markers were also analysed. Only 63 of the initially 115 literature-derived SSR markers were used for genotyping the primary F_1_ mapping population, because many of the remaining markers were not polymorphic in *Mb*j, a wild *M. baccata* genotype with a divergent genetic background compared with *M. domestica*. Of these 63 SSRs, 58 mapped successfully to the 17 linkage groups. Nearly all mapped SSRs corresponded well with the positions reported on the HiDRAS website (2025) and therefore remain suitable for comparison with previous and future *Malus* mapping studies, even though for some linkage groups only one SSR marker is present as shown in **Supplementary Table S4**. To further evaluate marker distribution, the physical positions of SNP markers of each chromosome were compared with the positions of the corresponding linkage groups. This analysis showed that SNPs typically started within the first few megabases and extended toward the chromosome ends, while gaps remain in some intermediate regions. One notable exception was LG 7, where the first mapped SNP marker was located at 31 Mb, and the linkage group itself measured only 19.1 cM. This suggests that the proximal region of chromosome 7 is a recombination-poor region in the studied population, likely resulting in the exclusion of markers up to 31 Mb during linkage map construction. Importantly, this linkage map allowed the detection of loci associated with powdery mildew resistance on LG 5 and LG 10.

Because powdery mildew in apple is caused by various pathogen strains (Urbanietz and Dunemann, 2005; Lesemann et al., 2004; Gañán-Betancur et al., 2021), phenotypic evaluation under natural, high-disease-pressure field conditions over multiple years can be considered a robust method to assess the durability and effectiveness of a resistance gene. Therefore, the result of this study, namely that *Plbj* was consistently detected in an F_1_ population from 2009–11 and 2023–24 under field conditions without fungicide application, assessed by two different persons with different assessment scales, exhibits the durability and heritability of this dominant haplotype-specific resistance. LOD scores on LG 10 exceeded the genome-wide significance threshold at every phenotyping time point. The percentage of explained phenotypic variance varied between the phenotyping time points, likely due to differences in disease pressure and weather conditions, but reached up to 74%. Since similarly high LOD scores (> 25) and explained phenotypic variances (> 65%) have been reported for major resistance genes against fire blight (Peil et al., 2007; Fahrentrapp et al., 2013; Broggini et al., 2014; Emeriewen et al., 2014, 2018), it can be hypothesised that *Plbj* also represents a major monogenic resistance gene. Therefore, the precise identification and delimitation of the genomic region harbouring *Plbj* is crucial. In this study, the candidate region could be narrowed down to a 3,287,286 bp interval through genetic mapping. Since this region has been sequenced (Pfeifer et al., 2026), an initial prediction of 29 potential resistance genes was possible, representing an important first step toward the functional analysis of *Plbj*. Although the precise cause of resistance at the *Plbj* locus remains unknown, it can be speculated that it is mediated by an NBS-LRR gene, as most of the 29 predicted genes contain domains characteristic of NBS-LRR proteins (Marone et al., 2013). According to the gene-for-gene hypothesis, these resistance genes are usually dominantly inherited, as observed for *Plbj*, and can specifically recognise corresponding *Avr* gene products from pathogens, thereby triggering a defence response (Flor, 1971; Marone et al., 2013). Future fine-mapping approaches using more recombinants, combined with the development of additional markers in the candidate region, are needed to further improve resolution and narrow down the locus. Moreover, the recent availability of the *Podosphaera leucotricha* genome will facilitate future research on the fungus itself as well as host-pathogen interactions (Gañán et al., 2020), especially once specific resistance genes in *Mb*j have been identified.

The strong effect of *Plbj* was clearly detectable in all years of the study. However, a few genotype-phenotype incongruences were observed. One genotype that carried the resistance marker alleles of the five newly develepod SSRs on LG 10 was nevertheless scored as susceptible, with scores of three and four at two field phenotyping time points in 2023 and 2024. As this genotype was scored as resistant in most years, a misclassification in those two years, possibly due to confusion with susceptible neighbouring trees, appears plausible. Conversely, three greenhouse-grown individuals from the 24230 population carried the resistance marker alleles of *Plbj*, but still showed noticeable powdery mildew symptoms in 2025. Possible explanations include mutations in the resistance gene itself, the presence of modifier genes suppressing resistance expression, or stress-induced susceptibility caused by limited plant spacing, reduced sunlight and partial spider mite infestation in the greenhouse. Furthermore, greenhouse conditions are markedly different from field conditions and have an influence on powdery mildew susceptibility (Jeger et al., 1986). Interestingly, it has already been described that genotypes with *Pl-m* appearing susceptible under artificial conditions still exhibit resistance in the field (Bus et al., 2010), suggesting that similar effects may explain these observations.

In addition to *Plbj*, a minor QTL on LG 5 HT2 was identified, showing chromosome-wide significance in three out of five scoring years. This QTL has not been previously reported by Dunemann and Schuster (2009). It remains unclear whether this locus represents a single gene or polygenic resistance. However, as the phenotypic variance explained by this locus was only up to 16%, compared to 74% for *Plbj*, and the LOD peak was noticeably flatter, it can be hypothesised that resistance at this locus is polygenic. Polygenic resistance is often more durable than monogenic major resistance genes (Parlevliet, 2002). Therefore, minor QTLs such as the one detected on LG 5 should not be underestimated, as they may contribute to the long-term stability of resistance and reduce the likelihood of resistance breakdown, which has not been reported so far for *Mb*j. Previous investigations have shown that QTLs for powdery mildew resistance on linkage groups 1, 8, 10, 14, and 17 are sometimes identified only in specific years, with phenotypic variation explained ranging from 5.1 to 19.5%, in contrast to stable QTLs on linkage groups 2 and 13, for which the phenotypic variation explained ranges from 7.5 to 27.4% across years (Calenge and Durel, 2006). In our study, a QTL peak on LG 5 was observed in all years, even when it did not consistently exceed the chromosome-wide significance threshold in interval mapping. This, together with a significant KW association, suggests that the underlying resistance effect was present across all years but varied in strength between years.

Molecular markers are nowadays crucial for marker-assisted selection, allowing selection based solely on genotypic information. For various powdery mildew resistance genes in apple, molecular markers have been reported (Markussen et al., 1995; Seglias and Gessler, 1997; Gardiner et al., 2003; James and Evans, 2004; Dunemann et al., 2007; Dunemann and Schuster, 2009; Bus et al., 2010; Luo et al., 2019; García-Gómez et al., 2024). However, some of these resistance genes, such as *Pl-1* (Krieghoff, 1995; Kellerhals et al., 2013) and *Pl-2* (Caffier and Laurens, 2005; Caffier and Parisi, 2007), have already been overcome and for others, like *Plbj*, the previously most closely linked marker was an AFLP/SCAR marker. In contrast, SSR and KASP markers are now more commonly used. In this study, we employed the published haplotype-resolved genome sequence of *Mb*j (Pfeifer et al., 2026), which facilitated the development of five SSR and two KASP markers linked to the resistance locus on LG 10 and one SSR linked to the resistance on LG 5. KASP markers are becoming increasingly important, as their analysis is relatively cheap and rapid, requiring only a real-time PCR machine rather than a costly capillary electrophoresis genetic analyser. The markers reported here can now be readily used in apple breeding programmes to efficiently select progenies with *Plbj* resistance. The potential presence of both a major monogenic resistance locus (*Plbj*) and a minor polygenic QTL in *Mb*j, both of which are dominantly inherited, makes this genotype highly attractive for breeding, as it may allow the combination of both types of resistance, each with its respective advantages, within a single donor genotype. Moreover, *Mb*j has previously been shown to exhibit resistance to apple scab (Gygax et al., 2004), several strains of fire blight (Vogt et al., 2013; Wöhner et al., 2018), and tolerance to *Diplocarpon coronariae* (Wöhner et al., 2021). Taken together, *Mb*j represents an exceptionally valuable donor for future apple resistance breeding, which is gaining in importance for sustainable apple production.

## Data Availability

The data presented in the study are deposited in the national center for biotechnology information (NCBI, https://www.ncbi.nlm.nih.gov/) repository, accession number PRJNA1402723.
