# Supplementary figures and images for: Genetic mapping, marker development, and identification of candidate genes for powdery mildew resistance in Malus baccata ‘Jackii’
Source: Front Plant Sci. 2026 Feb 12;16:1716290. doi: 10.3389/fpls.2025.1716290 (PMC12955710; doi:10.3389/fpls.2025.1716290)

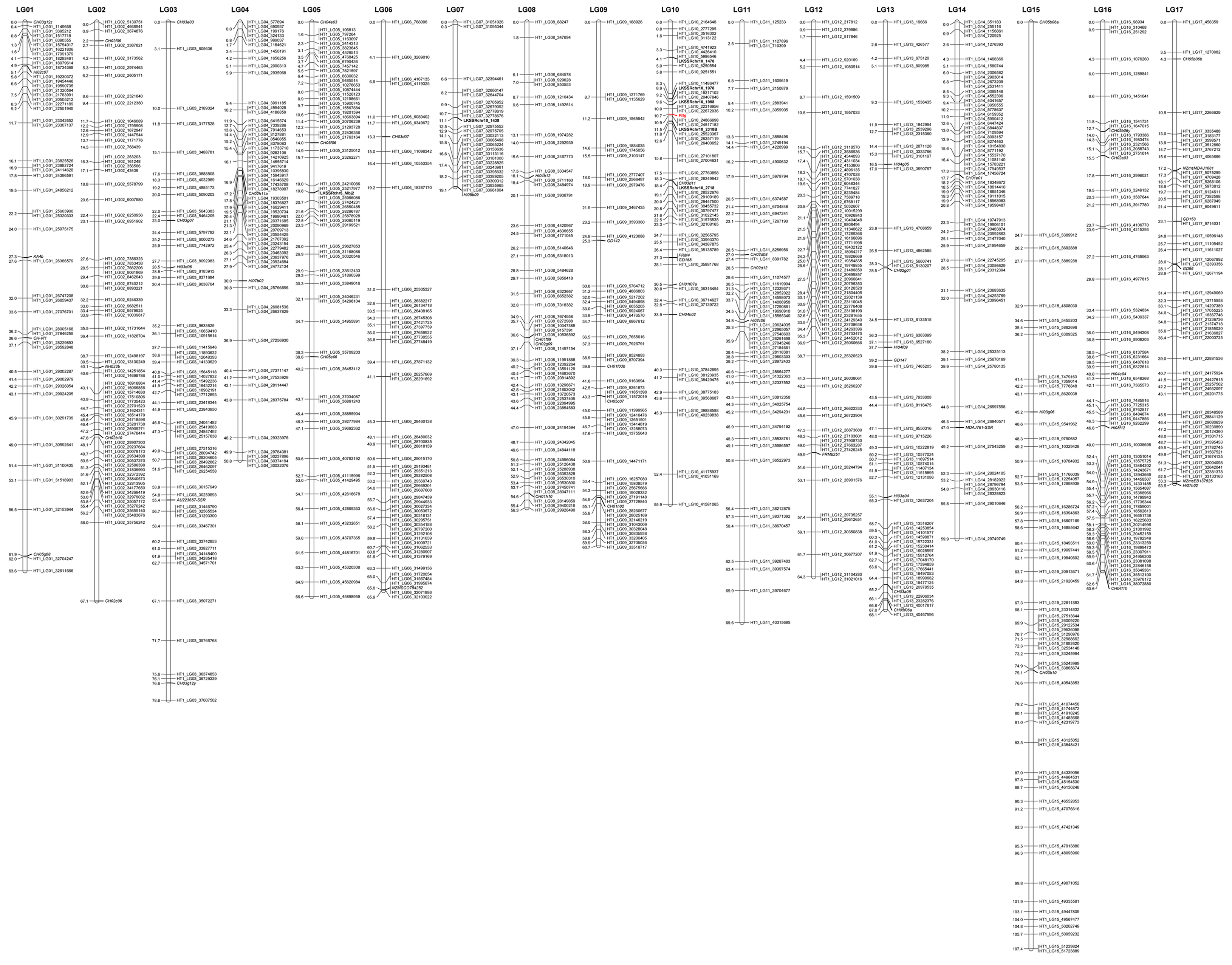

Supplement: Supplementary file 1 [file Image1.tif]
