# Supplementary material for: Genetic mapping, marker development, and identification of candidate genes for powdery mildew resistance in Malus baccata ‘Jackii’
Source: Front Plant Sci. 2026 Feb 12;16:1716290. doi: 10.3389/fpls.2025.1716290 (PMC12955710; doi:10.3389/fpls.2025.1716290)
Supplement: Supplementary file 2 [file Table1.docx]

**Supplementary information**

**Table S1.** Previously published SSR markers selected from the HiDRAS website (HiDRAS, 2025)

| **Reference** | **Marker names** |
| --- | --- |
| Celton et al., 2009 | NZmsCN943067, NZmsCO754252, NZmsEB137525, NZmsMDAJ1681 |
| Emeriewen et al., 2014 | FRM4 |
| Emeriewen et al., 2017 | FRMb251 |
| Hemmat et al., 2003 | GD153, GD158 |
| Hokanson et al., 1998 | GD96, GD142, GD147 |
| Liebhard et al., 2002 | CH01e01, CH01f03b, CH01f07a, CH01f09, CH01h02, CH01h10, CH02a03, CH02b03b, CH02b10, CH02c06, CH02d08, CH02d12, CH02f06, CH02g01, CH02g09, CH02h11a, CH03a08, CH03b10, CH03d07, CH03d11, CH03e03, CH03g07, CH03g12, CH04e03, CH04f10, CH04h02, CH05b06, CH05c07, CH05e06, Ch05f06, CH05g08 |
| Silfverberg-Dilworth et al., 2006 | AU223657-SSR, Hi01d05, Hi02c06, Hi02c07, Hi03a10, Hi03d06, Hi03e04, Hi03g06, Hi04b12, Hi04d02, Hi04e04, Hi04f09, Hi04g05, Hi05b09, Hi07b02, Hi07h02, Hi08f12, MDAJ761-SSR |
| Vinatzer et al., 2004 | CH-Vf1 |
| Yamamoto et al., 2002a | KA4b |
| Yamamoto et al., 2002b | NH033b |

**Table S2.** Newly developed SSR markers with primer sequences

| **Marker name** | **Forward primer sequence (5'-3')** | **Reverse primer sequence (5'-3')** |
| --- | --- | --- |
| LKSSRchr5_Mbj2 | CTTCTCCCTTGCTTGCTTCC | AGGGATCATGATACACTCGGT |
| LKSSRchr10_1438 | CGATTACAGAGACGGAGCGA | TTTATTGGCTGGGACGTCAC |
| LKSSRchr10_1478 | ACCACTACACCACAACCCAA | GGTTTCGGTGTTGGTTGTGA |
| LKSSRchr10_1978 | TTGGGTGAGGAGAGGGGTAT | ATCAGGTTTCGTCAGAGCCA |
| LKSSRchr10_1998 | ATTGGTTTGGGATGTCACGC | ACAAGAGATTGATCACTGGAGAA |
| LKSSRchr10_2318B | TCTCTCCCTTCCAATCCCAA | AGCCTCACTACTATTTAGCCAAT |
| LKSSRchr10_2718 | TGGAATGTTGTCTAATTAGGGCA | GTAACTATTGCTTTCCGGCCC |

**Table S3.** Overview of developed KASP markers

| **Marker name** | **FAM allele** | **HEX allele** | **Allele-specific forward primer 1 (5'-3')** | **Allele-specific forward primer 2 (5'-3')** | **Commone reverse primer sequence (5'-3')** |
| --- | --- | --- | --- | --- | --- |
| KASP_HT1_LG10_20042456 | C | T | GTGTAATGGGCTTGATCAACCAG | TCGTGTAATGGGCTTGATCAACCAA | CATAGTGATTAAATGATCTGAAGGGCCAA |
| KASP_HT1_LG10_23962775 | C | G | CAAAAATACCTTCAGAACAAGTTCTAGG | CAAAAATACCTTCAGAACAAGTTCTAGC | TTGACTTTKGGCATGACACTTTACTAGAAT |

**Table S4.** Number of markers and genetic length (cM) of the 17 linkage groups of *M. baccata* 'Jackii' haplotype 1

| **Linkage group** | **SNP marker** | **SSR marker** | **Length (cM)** |
| --- | --- | --- | --- |
| 1 | 47 | 5 | 63.6 |
| 2 | 70 | 4 | 67.1 |
| 3 | 58 | 5 | 78.6 |
| 4 | 58 | 2 | 50.8 |
| 5 | 62 | 4 | 66.6 |
| 6 | 53 | 2 | 65.9 |
| 7 | 24 | 2 | 19.1 |
| 8 | 55 | 4 | 56.3 |
| 9 | 51 | 4 | 60.7 |
| 10 | 50 | 10 | 55.9 |
| 11 | 53 | 3 | 69.6 |
| 12 | 69 | 1 | 64.3 |
| 13 | 51 | 7 | 68.1 |
| 14 | 61 | 2 | 59.9 |
| 15 | 65 | 3 | 107.4 |
| 16 | 67 | 5 | 63.6 |
| 17 | 54 | 6 | 53.5 |
| Total | 948 | 69 | 1071.0 |

**Table S5.** Summary of newly developed SSR markers linked to powdery mildew resistance

| **Marker** | **Linkage group** | **Predicted genomic position in haplotype 1 / 2** | **Expected PCR product size in bp of haplotype 1 / 2^a^** | **Observed size in fragment length analysis (bp)^a^** |
| --- | --- | --- | --- | --- |
| LKSSRchr5_Mbj2 | 5 | 25,400,896-25,400,994 / 25,183,631-25,183,741 | 99 / **111** | 99 / **111** |
| LKSSRchr10_1478 | 10 | 17,120,061-17,120,242 / 16,946,103- 16,946,288 | **182** / 186 | **177** / 181 |
| LKSSRchr10_1978 | 10 | 21,155,277-21,155,449 / 21,035,950- 21,036,126 | **175** / 179 | **176** / 180 |
| LKSSRchr10_1998 | 10 | 21,391,017-21,391,236 / 21,254,415- 21,254,632 | **220** / 218 | 215 / **217** |
| LKSSRchr10_2318B | 10 | 24,678,521 -24,678,663/ 24,412,181- 24,412,327 | **143** / 147 | **143** / 147 |
| LKSSRchr10_2718 | 10 | 28,170,148-28,170,275 / 27,895,197- 27,895,319 | **128** / 123 | 119 / **124** |

^a^ Resistance-associated alleles are shown in bold.

**Table S6.** Annotated genes in the region of interest in haplotype 1 of LG 10 in *M. baccata* 'Jackii'.

**Table S7.** Identified resistance gene candidates for *Plbj.*

**Figure S1.** Genetic linkage maps of haplotype 1 of *M. baccata* 'Jackii'. SSRs developed in this project are shown in bold, SSRs selected from the HiDRAS website (HiDRAS, 2025) in italics, and *Plbj* is indicated with a bold red italic label. For multilocus markers, a lowercase letter is appended to denote the specific locus.

For Table S6, S7 and Figure S1 please see separate supplementary files.
